# Teaching Global Health at the Frontlines: A multidisciplinary course in Peru presents basic concepts to students

**DOI:** 10.1371/journal.pmed.0040130

**Published:** 2007-06-05

**Authors:** Javier Villafuerte-Galvez, Walter H Curioso

## Abstract

The authors discuss a unique project to teach medical students in Peru about global health, which included a trip to the mining town of La Oroya at 15,610 feet above sea level in the Peruvian Andes.

Global health is a challenge to define, and even more of a challenge to improve. A broad definition of global health is given by the United States Institute of Medicine as “health problems, issues, and concerns that transcend national boundaries, may be influenced by circumstances or experiences in other countries, and are best addressed by cooperative actions and solutions” [1].

Most medical students in rich countries think that there should be more teaching on global health issues [2], and many have led the way in calling for global health to be included in their curriculum [3]. Little is known, however, about the experiences of teaching and learning about global health in medical schools in developing countries, which are in the frontlines of global health problems.

The experience of learning about global health in a developing country such as Peru is best described by the motto of the Gorgas International Post-Graduate Course taught at the Universidad Peruana Cayetano Heredia (UPCH) every year: “Teaching tropical medicine in the tropics” [4]. UPCH and its collaborators have long recognized that infectious diseases training and research needs to be both hands-on and conducted in the actual context where public health challenges are encountered, enabling realistic evaluation and problem solving [4].

This essay discusses, from the point of view of a fourth-year medical student and a research professor, a one-week course on global health held at UPCH, which included a one-day road trip to the mining town of La Oroya near Ticlio, 15,610 feet above sea level in the Peruvian Andes.

## Basic Concepts in Global Health: A Peruvian Experience

As part of the activities of UPCH's Global Health Peru Program (http://www.globalhealthperu.org), an elective course entitled “Basic Concepts in Global Health” was organized in July 2006. The goals of this one-week elective course were: (1) to bring together students from diverse schools within UPCH and two universities in Peru and introduce them to the basic concepts of global health and (2) to encourage these students to share information and create an ongoing multidisciplinary and interdisciplinary dialogue.

Course participants were undergraduate students from the schools of medicine, public health, sciences, dentistry, and veterinarian medicine. Small group sessions were organized in order to allow interaction between all students, who worked side by side through research tutorials leading to the development of a group research project. Sessions were led by a mix of Peruvian faculty, officials from global health organizations based in Peru, and representatives from the Peruvian Ministry of Health.

During the last day of the course, all the students traveled to La Oroya to explore the impact of social, cultural, and geographic factors upon health in a global context.

## Lessons Learned

The main lesson I (JV-G) learned in this course was to take a broader look at every health problem I face. I need to not only take into account the complexity of the individual, but to pay attention to their interplay with the community at the local and global levels. Other important lessons are described below.

### Local and global economics

The economy of the central Andes went from a traditional, local, exchange-based economy to an economy based on mineral extraction and oriented to the global market. I witnessed how globalization and mining brought in new technologies, which gave the communities more access to the global market but also caused great disturbance in people's lifestyles, their health, and their ancestrally established economic networks.

### Poverty and employment

**Figure 1:**
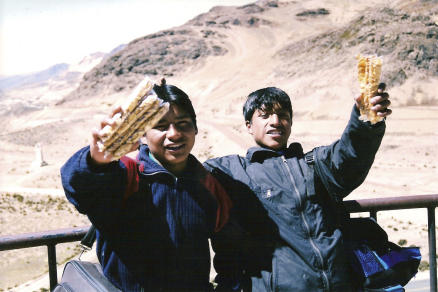
*Haberos:* Snack Sellers in Ticlio (Photo: Javier Villafuerte-Galvez)

The switch in economic activities doesn't seem to have provided enough opportunities for education and employment. I met young men my age whose main activity was to sell toasted fava bean snacks *(“habas tostadas”)* to tourists (Figure 1). They told me they had the “desire to study, do something with their lives,” but said that this was a remote dream.

### Environmental health

La Oroya is a mining town in the Peruvian Andes about 60 kilometers east of Ticlio, and it has been exposed for decades to the toxic emissions (heavy metals and sulfur dioxide) from a metallurgical plant that produces mainly lead, silver, and gold. These emissions are largely responsible for the dangerously high blood lead levels found in the children of this community (33.6 μg/dl) [5]. Lead is not only harmful to cognitive development, but is toxic to the kidneys, lymphoid organs, and central nervous system [6].

Mining has had a huge impact upon the community and has even changed the shape of the region's landscape through the accumulation of mining debris, called *relaves*, which are sometimes as high as hills. Mining has also caused the destruction of native vegetation through acid rain, caused by sulfur dioxide emissions that exceed the World Health Organization's emissions standards tenfold [7].

Unfortunately, even after seeing these shocking results in 1999, the Peruvian Ministry of Health has taken little action to treat the children of La Oroya, limit exposure to these emissions, or educate the public about the health risks [8].

### Unequal access to health services

Health-care services are few, and most health-care centers do not have the capability to treat complicated cases. The capacity to refer a patient to a more complex health facility is one of the cornerstones of primary health care. If I (JV-G) were the doctor in a rural primary care facility and I diagnosed, for example, hepatic hydatid disease in a woman who earned less than a dollar a day, what would be the odds of me getting her to a regional or national reference center for surgical treatment?

Because of the lack of access to formal health care, some communities have coped by using the only health care available to them: traditional medicine, a practice that should be embraced by outside physicians as part of an overall medical solution.

### Migration

During the last 40 years, thousands of migrants from the central Andes (and all over the country) have fled to Lima to seek better opportunities. The city now has 8 million residents. Almost 40% of the residents of Lima are poor and are concentrated in peripheral urban conglomerates, called *“conos,”* which contain almost two-thirds of the population of the city [9]. These migrants mainly attend health facilities that lack the physical and human resources to adequately meet patients' health needs. While the recent introduction of MAIS (*Modelo de Atención Integral en Salud*, or Integral Health Care Model) by the Ministry of Health [10] is enhancing the capacity of regions and communities to develop local health policy, Peruvian health policy is still mainly determined in central Lima.

### Access to information

While most Peruvians access the Internet through a Peruvian version of Internet cafes called *“cabinas públicas”* [11], searching for health information on the Internet could be a difficult experience for a rural health-care worker. Resources may not necessarily be relevant or appropriate, while high-quality, peer-reviewed articles may not be available due to costs (per article or for a subscription). Health-care workers may also find searching to be time consuming and difficult, and, most frustrating, information may not be in their language.

## Evaluation of the Course

I (WC) found that the students were very enthusiastic about the course. In the final evaluation, 97% of the respondents (19/20) rated the course as very good/excellent, 100% rated the usefulness of the course as very good/excellent, and 90% (18/20) would definitely recommend the course to their peers. In addition, some students are now in discussion with mentors about implementation or further funding of their research projects. Due to the positive response from the course, we are organizing the next course in July 2007 with the participation of international medical students.

## Conclusion

La Oroya, like many poor communities, has a lot to teach students about the challenges of global health. Global health problems are complex and can't be solved by a “magic bullet,” such as a simple vaccine or medicine. The residents of La Oroya suffer from poverty, the effects of mining, and unequal access to health care, all which exacerbate their health problems.

Global health problems can't be solved by concerned physicians alone; they also require government policies. We can't solve the problems of La Oroya without looking at how globalization and poverty have affected the life of the community.

## Supplementary Information

Alternative Language Text S1 Spanish translation of the article by JV-G and WC (63 KB DOC).

## Abbreviations

UPCH: Universidad Peruana Cayetano Heredia
