# Supplementary material for: Teaching Global Health at the Frontlines: A multidisciplinary course in Peru presents basic concepts to students
Source: PLoS Med. 2007 Jun 5;4(6):e130. doi: 10.1371/journal.pmed.0040130 (PMC1885452; doi:10.1371/journal.pmed.0040130)
Supplement: Supplementary file 1 [file pmed.0040130.sd001.doc]

### FORO ESTUDIANTIL

Enseñando Salud Global en el Campo de Acción

*Un curso multidisciplinario en Perú presenta conceptos básicos a estudiantes*

**Javier Villafuerte-Galvez, Walter H. Curioso^*^**

Javier Villafuerte-Galvez es un estudiante del cuarto año de medicina de Facultad de Medicina y es Presidente de la Sociedad Científica de Estudiantes de “SOCEMCH”, Universidad Peruana Cayetano Heredia (UPCH), Lima, Perú. Walter H. Curioso es profesor investigador en el Programa Salud Global Perú en la Facultad de Salud Pública y Administración, y en la Facultad de Medicina, UPCH, Lima, Perú.

**Financiamiento**: El Programa Salud Global Perú de la UPCH ([http://www.globalhealthperu.org](http://www.globalhealthperu.org/)) está financiado por un fondo del Fogarty International Center/National Institutes of Health (5R25TW007490).

**Conflictos de Interés:** Los autores han declarado que no existen conflictos de interés.

*A quien la correspondencia debe ser dirigida: E-mail: [wcurioso@u.washington.edu](mailto:wcurioso@u.washington.edu)

Es un reto definir a la salud global, lo es aún más mejorarla. El Instituto de Medicina de los Estados Unidos la define de forma amplia como los “problemas y temas relacionados la salud que trascienden las fronteras de una nación, que pueden estar influenciados por circunstancias o experiencias de otros países y que son enfrentados mejor mediante acciones y soluciones cooperativas” [1]

La mayoría de los estudiantes de medicina en países desarrollados consideran que debería haber más enseñanza respecto a problemas de salud global [2], y en numerosos países desarrollados han sido los estudiantes quienes han liderado los pedidos de inclusión de la salud global en sus planes de estudios [3]. Poco se sabe, sin embargo, sobre experiencias de enseñanza y aprendizaje en salud global en escuelas de medicina de los países en desarrollo que se encuentran en el campo de acción de los problemas de salud global.

Aprender sobre salud global en un país en desarrollo como Perú se encuentra bien descrito por el lema del Curso Internacional de Post-Grado Gorgas que se enseña en la Universidad Peruana Cayetano Heredia (UPCH) cada año por dos meses: “Enseñando medicina tropical en los trópicos” [4]. La UPCH y sus colaboradores han reconocido por buen tiempo que el entrenamiento en enfermedades infecciosas necesita ser práctico así como la investigación ha de ser conducida en el contexto mismo en el cual se enfrentan los problemas de salud pública; permitiendo la resolución de problemas así como una evaluación realista. Este ensayo discute, desde el punto de vista de un estudiante de medicina del cuarto año y de un profesor investigador, un curso de una semana sobre salud global que tuvo lugar en la UPCH, el cual incluyó un viaje de un día a la ciudad minera de La Oroya que se encuentra cercana a Ticlio, a 4,843 metros sobre el nivel del mar en los Andes Peruanos.

# Conceptos Básicos en Salud Global:

# Una experiencia peruana

Como parte de las actividades del Programa Salud Global Perú de la UPCH, se organizó un curso electivo titulado “Conceptos Básicos de Salud Global” en Julio del 2006. Los objetivos de este curso electivo de una semana fueron: (1) reunir a estudiantes de diversas facultades dentro de la UPCH y de dos universidades peruanas para presentarles los conceptos básicos de salud global así como (2) motivar a estos estudiantes a compartir información y crear un diálogo multi e interdisciplinario continuo.

Los participantes del curso fueron estudiantes de pregrado de las facultades de medicina, salud pública, ciencias, estomatología y medicina veterinaria. Se organizaron sesiones en pequeños grupos con el fin de interactuar con todos los estudiantes; y se desarrollaron tutoriales de investigación hasta la elaboración de un proyecto de investigación grupal. Las sesiones fueron dirigidas por un grupo de profesores peruanos, oficiales de organizaciones de salud global establecidas en Perú y representantes del Ministerio de Salud.

Durante el último día del curso, todos los estudiantes viajaron a La Oroya a explorar el impacto de los factores sociales, culturales y geográficos sobre la salud dentro de un contexto global.

# Lecciones Aprendidas

La principal elección que yo (JV-G) aprendí en este curso fue tener una visión más amplia de cada problema al frente del cual me encuentre. Tengo que tomar en cuenta no sólo la complejidad del individuo, pero tambien prestar atención al rol que juega éste dentro de su comunidad a escala local y global. Otras lecciones importantes incluyen:

**Economiza local y la interacción con la economia global**. La economía de los Andes centrales se ha transformado de ser una economía local tradicional, basada en el intercambio hacia una economía basada en la extracción de minerales y orientada al mercado global. Fui testigo de cómo la globalización y la minería han traído nuevas tecnologías, las cuales han dado a las comunidades más acceso al mercado global pero han causado igualmente grandes perturbaciones en los estilos de vida de las personas, en su salud, y en sus redes económicas establecidas ancestralmente.

**Pobreza y empleo**. El cambio en las actividades económicas no parece haber traído suficientes oportunidades para la educación y el empleo. Conocí a jóvenes de mi edad cuya principal actividad era vender habas tostadas a turistas (Figura 1). Ellos me contaron que tenían el “deseo de estudiar, hacer algo con sus vidas,” pero dijeron que era un sueño remoto.

**Salud ambiental.** La Oroya es una ciudad minera en los Andes Peruanos situada aproximadamente a 60 km al este de Ticlio y ha sido expuesta por décadas a las emisiones toxicas (metales pesados y dióxido de azufre) de una planta metalúrgica que produce principalmente cobre, plata, y oro. Estas emisiones son las grandes responsables de los peligrosos altos niveles de plomo en sangre encontrados en niños de esta comunidad (33.6μg/dL) [5]. El plomo no es sólo dañino para un adecuado desarrollo mental, se conoce que también causa severa toxicidad a los riñones, los órganos linfoides, y el sistema nervioso central [6].

La minería ha tenido un gran impacto en la comunidad e incluso ha cambiado la forma del paisaje de la región a través de la acumulación de relaves (desperdicios productos de la minería), los cuales son algunas veces tan altos como una colina. La minería también ha causado la destrucción de la vegetación nativa por las lluvias de ácido causados por las emisiones de dióxido de azufre que exceden los estándares de la OMS hasta en 10 veces [7].

Desafortunadamente, incluso después de haber sido publicados estos resultados alarmantes en 1999, el Ministerio de Salud del Perú ha tomado poca acción para tratar a los niños de La Oroya, para limitar la exposición, o educar al público acerca de estos riesgos para la salud [8].

**Inequidad de acceso a los servicios de salud.** Los servicios para la atención de salud son limitados, y la mayoría de los establecimientos de salud no tienen la capacidad de atender casos complicados. La capacidad para referir un paciente hacia un establecimiento salud de mayor complejidad es una de las piedras angulares de la atención primaria en salud. Si yo (JV-G) fuera un médico en un establecimiento de salud primario rural y diagnosticara, por ejemplo, una enfermedad hidatídica hepática en una mujer que gana menos de un dólar por día; ¿Cuáles son las probabilidades de trasferirla a un centro de referencia regional o nacional para que reciba tratamiento quirúrgico?

Debido a la falta de acceso a los centros de salud formalmente establecidos, algunas comunidades han optado por utilizar el único servicio de salud al que tienen acceso: la medicina tradicional, una práctica que debería ser adoptada por los médicos que practican en zonas rurales, como parte de una solución médica integral.

**Migración.** Durante los últimos 40 años, miles de migrantes han viajado desde los Andes centrales (y desde todas partes del país) a Lima para buscar mejores oportunidades. La ciudad de Lima ahora tiene 8 millones de residentes. Casi el 40% de los residentes de Lima son pobres y están concentrados en conglomerados urbanos periféricos llamados “*conos,*” los cuales albergan casi a los dos tercios de la población de la ciudad de Lima [9]. Estos migrantes acuden principalmente a establecimientos de salud sobrecargados que muchas veces no disponen de los recursos tanto humanos como físicos para atender adecuadamente las demandas de salud de la población. Mientras la reciente introducción del Modelo de Atención Integral en Salud (MAIS) por el Ministerio de Salud [10] está reforzando las capacidades de las regiones y las comunidades para desarrollar políticas de salud locales, las políticas de salud peruana todavía están determinada en la ciudad de Lima.

**Acceso a la información**: Mientras la mayoría de peruanos acceden al Internet desde cabinas públicas (una version peruana de los “Internet cafés” [11], para un trabajador de salud rural, la búsqueda de información en salud puede convertirse en una experiencia frustrante. Los recursos pueden no ser necesariamente relevantes o apropiados, estos recursos (específicamente artículos de alta calidad y revisados por pares) pueden no estar disponibles debido a los costos(por artículo o por suscripción), la búsqueda puede tomar mucho tiempo y ser difícil, y lo más frustrante, la información puede no estar en su propio lenguaje.

**Evaluación del Curso**

Encontré (WC) que los participantes estuvieron muy entusiasmados respecto al curso. Durante la evaluación final, 97% de los que respondieron (19/20) evaluaron el curso como muy bueno/excelente, 100% de los que respondieron evaluaron la utilidad del curso como muy bueno/excelente, y18 (90%) de los que respondieron recomendarían (“definitivamente sí”) este curso a sus colegas. Adicionalmente, algunos de los proyectos de investigacion de los estudiantes del curso están siendo discutidos con los asesores para ser implementados o para buscar financiamiento. Debido a la experiencia positiva del primer curso, estamos organizando el siguiente curso en Julio del 2007 con la participación de estudiantes de medicina internacionales.

# Conclusión

Una de las lecciones más importantes fue comprender los desafíos de la salud global al visitar una comunidad minera pobre en Perú. . Los problemas en salud global son complejos y no pueden resolverse con una simple vacuna. Los residentes de La Oroya sufren de pobreza, efectos de la minería, y de inequidad en el acceso a los servicios de salud, todos ellos exacerban los problemas de salud. Si solo uno de esos factores mejorara, la salud de la población podría mejorar. Los problemas en salud global no pueden resolverse sólo con médicos preocupados; requiere también de políticas de gobierno y más atención pública al problema. Nosotros no podemos resolver los problemas de La Oroya sin prestar atención a como la globalización y la pobreza ha afectado sus vidas. Esta es una importante lección para médicos en formación y trabajadores de salud y es el porqué se necesitan más cursos prácticos en salud global.

**Agradecimientos**: Nos gustaría agradecer a Patricia Garcia, MD, MPH directora e investigadora principal del Programa Salud Global Perú de la UPCH, y a Ciro Maguiña, MD del Instituto de Medicina Tropical “Alexander von Humboldt,” quienes organizaron y condujeron el viaje a La Oroya. También agradecemos a Bobbi Nodell, MA, por la asistencia editorial.

# Referencias

[1] Institute of Medicine (1997) America's Vital Interest in Global Health. Washington, DC: National Academy Press.

[2] Edwards R, Rowson M, Piachaud J (2001) Teaching international health issues to medical students. Med Educ 35:807-8.

[3] Bateman C, Baker T, Hoornenborg E, Ericsson U (2001) Bringing global issues to medical teaching. Lancet 358:1539-42.

[4] Freedman DO, Gotuzzo E, Seas C, Legua P, Plier DA, Vermund SH, Casebeer LL (2002) Educational programs to enhance medical expertise in tropical diseases: the Gorgas Course experience 1996-2001. Am J Trop Med Hyg 66:526-32.

.	[5] MINSA (1999) Estudio de Plomo en Sangre en una Población Seleccionada de La Oroya, Ministerio de Salud. Available: <http://www.aida-americas.org/templates/aida/uploads/docs/DIGESA_blood_Pb_Main.pdf>. Accessed 9 January 2007.

[6] Center for Disease Control and Prevention (2005) CDC’s Third National Report on Human Exposure to Environmental Chemicals. Available:

<http://www.cdc.gov/exposurereport/pdf/factsheet_lead.pdf>. Accessed 9 January 2007.

[7] Center for Disease Control and Prevention (2005). Development of an integrated intervention plan to reduce exposure to lead and other contaminants in the mining center of La Oroya, Peru. Centers for Disease Control and Prevention National Center for Environmental Health/ Agency for Toxic Substances and Disease Registry Division of Emergency and Environmental Health Services. Available: <http://www.cdc.gov/nceh/ehs/Docs/la_oroya_report.pdf>. Accessed 9 January 2007.

[8] Interamerican Association for Environmental Defense (2006) Interamerican Association for Environmental Defense. Available: <http://www.aida-americas.org/aida.php?page=13&lang=en>. Accessed 9 January 2007.

[9] National Institute of Statistics and Informatics (2005) National Household Survey. Results by levels of poverty. [Encuesta Nacional de Hogares – ENAHO]. Lima: Instituto Nacional de Estadística e Informática.

[10] MINSA (2006) Modelo de Atención Integral en Salud. Available: <http://www.minsa.gob.pe/portal/Servicios/SuSaludEsPrimero/MAIS/mais.asp> Accessed 9 January 2007.

[11] Curioso WH, Blas M, Nodell B, Alva I, Kurth AE. Opportunities for providing Web-based interventions for sexually transmitted infection prevention in Peru’s Internet cafes. PLoS Medicine. In press.
